# Treatment with GLP-1 receptor agonists is associated with significant weight loss and favorable headache outcomes in idiopathic intracranial hypertension

**DOI:** 10.1186/s10194-023-01631-z

**Published:** 2023-07-18

**Authors:** Nik Krajnc, Bianca Itariu, Stefan Macher, Wolfgang Marik, Jürgen Harreiter, Martin Michl, Klaus Novak, Christian Wöber, Berthold Pemp, Gabriel Bsteh

**Affiliations:** 1 Department of Neurology, Medical University of Vienna, Waehringer Guertel 18–20, 1090 Vienna, Austria; 2 Comprehensive Center for Clinical Neurosciences, Medical University of Vienna, & Mental Health, Vienna, Austria; 3 Department of Internal Medicine III, Division of Endocrinology, Medical University of Vienna, Vienna, Austria; 4 Department of Neuroradiology and Musculoskeletal Radiology, Medical University of Vienna, Vienna, Austria; 5 Department of Ophthalmology, Medical University of Vienna, Vienna, Austria; 6 Department of Neurosurgery, Medical University of Vienna, Vienna, Austria

**Keywords:** Idiopathic intracranial hypertension, Weight loss, Glucagon-like peptide-1, Headache, Visual worsening

## Abstract

**Background:**

In idiopathic intracranial hypertension (IIH), sustained weight loss is the main pillar in modifying disease course, whereby glucagon-like peptide-1 receptor agonists (GLP-1-RAs) could present an attractive treatment option.

**Methods:**

In this open-label, single-center, case–control pilot study, patients with IIH (pwIIH) and a body mass index (BMI) of ≥ 30 kg/m^2^ were offered to receive a GLP-1-RA (semaglutide, liraglutide) in addition to the usual care weight management (UCWM). Patients electing for UCWM only served as a control group matched for age-, sex- and BMI (1:2 ratio). The primary endpoint was the percentage weight loss at six months (M6) compared to baseline. Secondary endpoints included the rate of patients with a weight loss of ≥ 10%, monthly headache days (MHD), the rate of patients with a ≥ 30% and ≥ 50% reduction in MHD, visual outcome parameters, and adverse events (AEs).

**Results:**

We included 39 pwIIH (mean age 33.6 years [SD 8.0], 92.3% female, median BMI 36.3 kg/m^2^ [IQR 31.4–38.3]), with 13 patients being treated with GLP-1-RAs. At M6, mean weight loss was significantly higher in the GLP-1-RA group (–12.0% [3.3] vs. –2.8% [4.7]; *p* < 0.001). Accordingly, weight loss of ≥ 10% was more common in this group (69.2% vs. 4.0%; *p* < 0.001). Median reduction in MHD was significantly higher in the GLP-1-RA group (–4 [–10.5, 0.5] vs. 0 [–3, 1]; *p* = 0.02), and the 50% responder rate was 76.9% vs. 40.0% (*p* = 0.04). Visual outcome parameters did not change significantly from baseline to M6. Median reduction in acetazolamide dosage was significantly higher in the GLP-1-RA group (–16.5% [–50, 0] vs. 0% [–25, 50]; *p* = 0.04). AEs were mild or moderate and attributed to gastrointestinal symptoms in 9/13 patients. None of the AEs led to premature treatment discontinuation.

**Conclusions:**

This open-label, single-center pilot study suggests that GLP-1-RAs are an effective and safe treatment option for achieving significant weight loss with a favorable effect on headache, leading to reduced acetazolamide dosage in pwIIH.

**Graphical Abstract:**

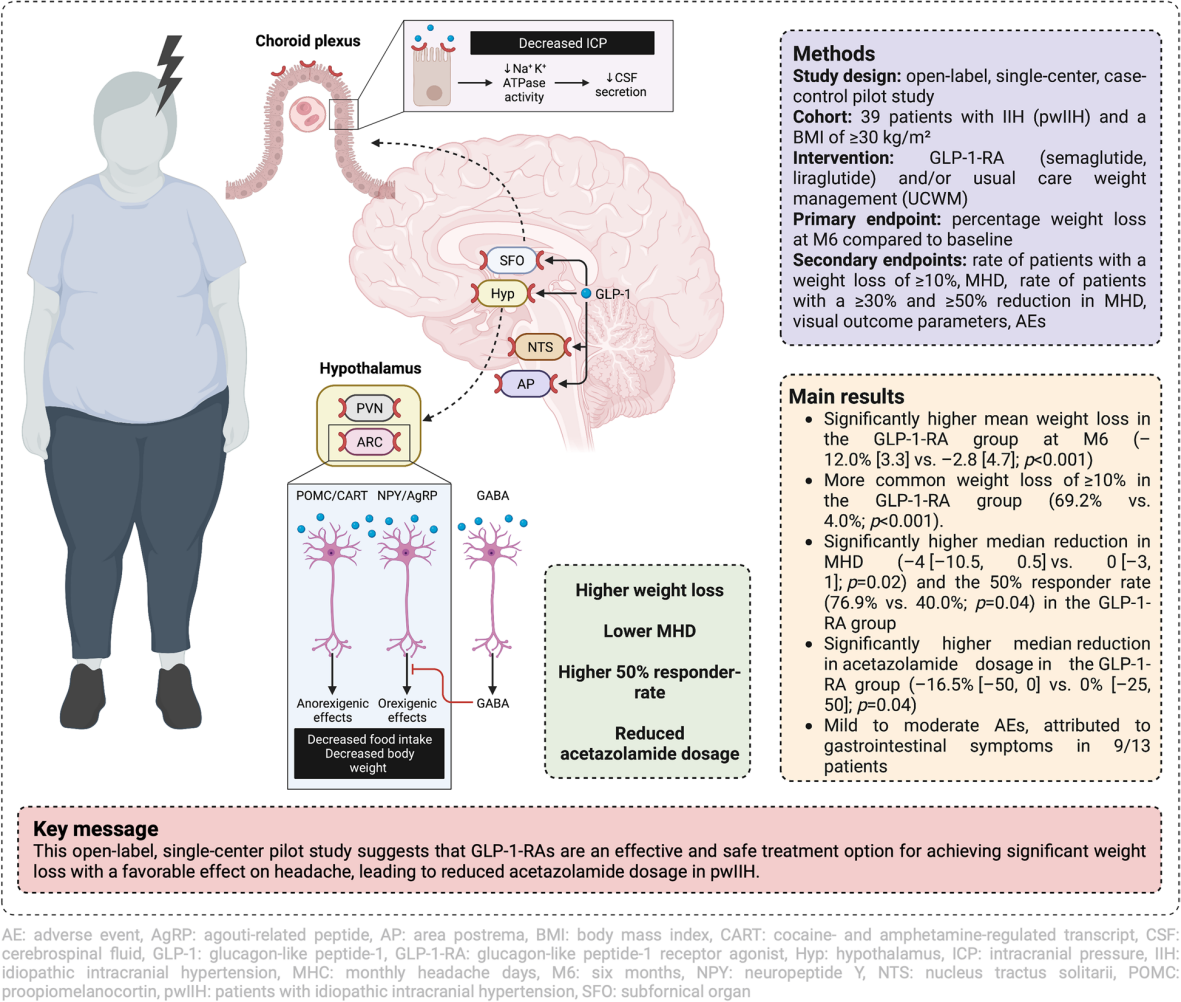

**Supplementary Information:**

The online version contains supplementary material available at 10.1186/s10194-023-01631-z.

## Background

Idiopathic intracranial hypertension (IIH) is a debilitating condition characterized by raised intracranial pressure causing chronic headaches and papilledema with the risk of permanent visual loss, which most commonly occurs in young obese women [1]. Its incidence is increasing with growing obesity rates worldwide [2], with only modest weight gain being associated with an increased risk of developing IIH or experiencing a relapse after remission [3]. In return, a reduction in body weight of 10% or more often leads to disease remission [4]. Thus, body weight is the main modifiable factor associated with the development of IIH, and weight loss interventions present the most effective approach in modifying the disease course of IIH [5, 6].

**Fig. 1:**
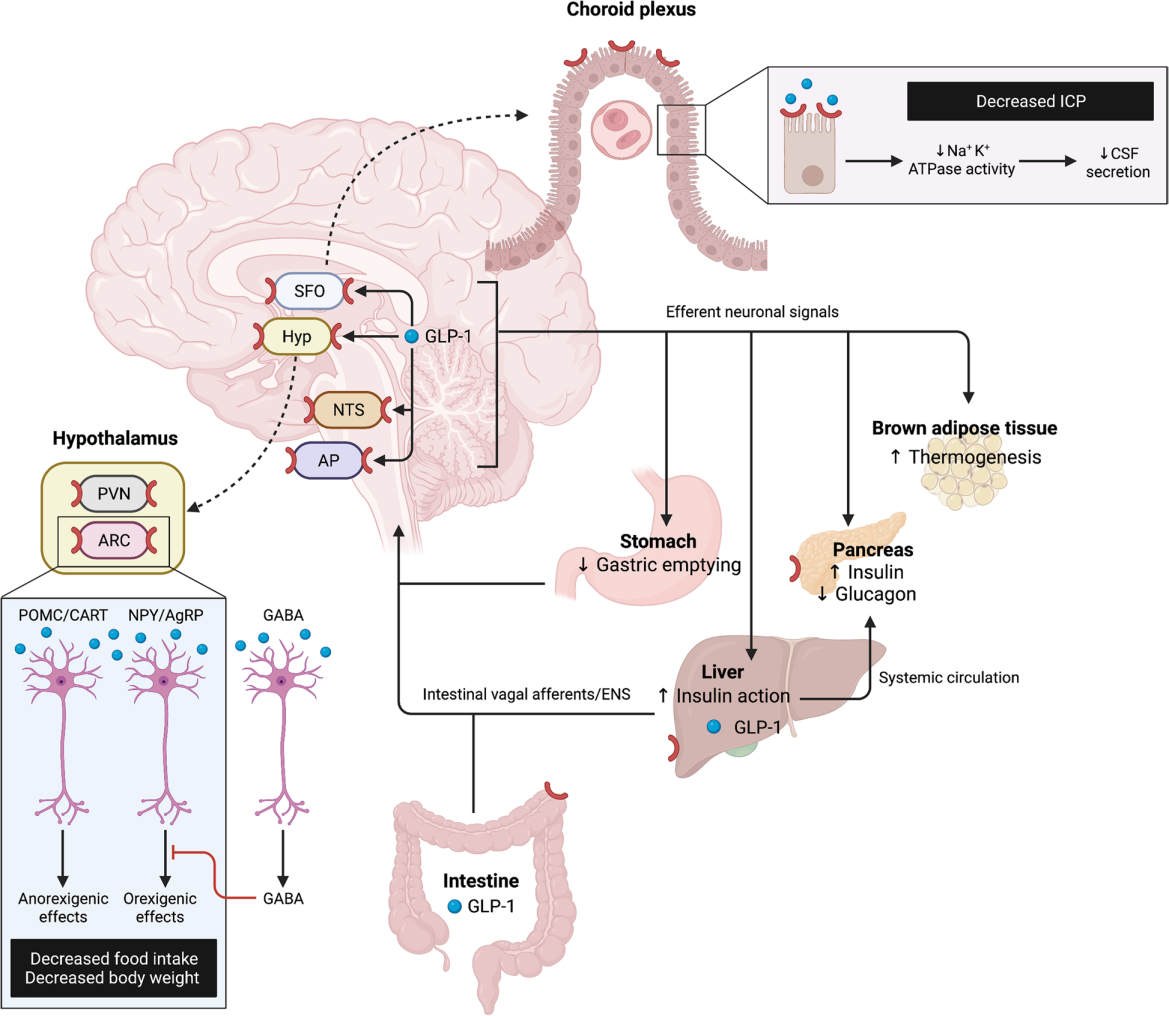
GLP-1 is secreted from enteroendocrine cells where it activates intestinal vagal afferents, located in the gut and portal circulation, further activating GLP-1-producing neurons in the nucleus tractus solitarii. These neurons project to several food-regulating areas, including the ventral tegmental area, the nucleus accumbens and the hypothalamus. There, GLP-1 directly activates POMC/CART neurons and indirectly inhibits, via GABAergic transmission, the NPY/AgRP neurons, which collectively results in signals reducing food intake. Efferent pathways, which originate in the brain stem, subsequently signal to peripheral organs to close the loop of feeding behavior and glucose metabolism regulation. GLP-1 receptors are also expressed on the choroid plexus epithelial cells, where the binding of GLP-1 reduces Na + K + ATPase activity, leading to decreased CSF secretion and consequently decreased ICP. Created with BioRender.com. AgRP: agouti-related peptide, AP: area postrema, CART: cocaine- and amphetamine-regulated transcript, CSF: cerebrospinal fluid, ENS: enteric nervous system, GLP-1: glucagon-like peptide-1, Hyp: hypothalamus, ICP: intracranial pressure, NPY: neuropeptide Y, NTS: nucleus tractus solitarii, POMC: proopiomelanocortin, SFO: subfornical organ

Glucagon-like peptide-1 (GLP-1) is a peptide hormone produced by enteroendocrine cells at low basal levels, but also by neurons in the caudal medulla [7]. It has potent effects on blood glucose by either stimulating glucose-induced insulin release or inhibiting glucagon secretion [8, 9], and suppresses appetite via receptors in the ventral tegmental area, the nucleus accumbens and the hypothalamus (Fig. 1) [10–13].

GLP-1 receptor agonists (GLP-1-RA) were first developed for the treatment of type-2 diabetes, but have been recently approved for treatment of obesity. Anti-obesity drug therapy can be offered as an adjunct to a reduced-calorie diet and increased physical activity for weight management in adult patients with an initial body mass index (BMI) of ≥ 30 kg/m^2^ or 27–30 kg/m^2^ in the presence of at least one weight-related comorbidity, e.g., dysglycemia, hypertension, dyslipidemia, or obstructive sleep apnea. As non-interventional approaches are often frustrating and bariatric surgery is an effective yet invasive procedure, GLP-1-RAs present an attractive treatment option in patients with IIH (pwIIH). Besides, GLP-1 receptors are also expressed in the human choroid plexus, and the GLP-1-RA exenatide was reported to reduce intracranial pressure in a rodent model as well as in a pilot study on 15 pwIIH [14, 15].

Thus, we aimed to study the effect of GLP-1-RAs on weight management, as well as on headache and visual outcomes in pwIIH.

## Methods

### Patients and definitions

Starting in March 2022, patients with IIH and BMI ≥ 30 kg/m^2^ were routinely offered to receive a GLP-1-RA (semaglutide, liraglutide) in addition to the usual care weight management (UCWM) consisting of dietary counselling and non-supervised physical exercise per treatment algorithm at the interdisciplinary IIH center at the Medical University of Vienna. We prescribed semaglutide or liraglutide in accordance with Austrian reimbursement regulations. Most of the patients decided for semaglutide for its weekly administration and higher efficacy in achieving weight loss compared to liraglutide [16]. Treatment with semaglutide was initiated at 0.25 mg per week and escalated to the maximum tolerated dose or up to 2 mg per week over 16 weeks. Semaglutide with the maximum dose of 2.4 mg (Wegovy®) was not available in Austria at the time of enrollment. Liraglutide, initiated at 0.6 mg per day, was escalated to the maximum tolerated dose or up to 3.0 mg per day over 4 weeks. Treatments were administered subcutaneously using a multidose pen injector. Medication for IIH with acetazolamide, topiramate and/or furosemide was continued independently of the treatment with GLP-1-RAs.

For this study, we used the Vienna Idiopathic Intracranial Hypertension (VIIH) database, a cohort comprising 151 patients with definite IIH according to modified Friedman criteria [12]. Details of the VIIH database are described elsewhere [13]. In brief, data are collected retrospectively at first visit and prospectively whenever the patient returns for scheduled follow-up (at least every 3 months) or unscheduled visits with neurologists and neuro-ophthalmologists specialized in IIH.

In a case–control design, patients electing to receive GLP-1-RA therapy in addition to UCWM were included based on the following inclusion criteria: definite IIH according to the modified Friedman criteria [17], BMI $\ge$ 30 kg/m^2^, and a follow-up of $\ge$ 6 months. As a control group, pwIIH electing for UCWM only were matched for age-, sex- and BMI in a 1:2 ratio.

The following data were obtained at baseline as well as three and six months (M3, M6) after initiation of the treatment with a GLP-1-RA + UCWM or UCWM alone: body weight, monthly headache days (MHD) recorded in a headache diary, and ophthalmological assessment including visual acuity, perimetry, fundoscopy, optical coherence tomography (OCT) and ocular ultrasonography. In addition, we recorded dosages of the GLP-1-RA and of IIH medication, adherence to the GLP-1-RA treatment and adverse events (AEs) at M3 and M6.

Best-corrected visual acuity was assessed using Sloan charts at distance after subjective refraction. Results were given in logarithm of the minimum angle of resolution (logMAR). Meaningful change was defined as ≥ 0.2 logMAR [18].

For perimetry, we performed automated visual field testing (Humphrey Field Analyzer, Carl Zeiss Meditec, Jena, Germany) using 30–2 Swedish Interactive Threshold Algorithm (SITA) standard protocols, quantifying the mean deviation in decibels (dB) of all test locations compared to age-matched controls and defining abnormal perimetry as a mean deviation lower than -2 dB.

Fundoscopy included assessment of absence or presence of papilledema and secondary optic atrophy. We used the Frisén staging scale to rate papilledema severity, categorizing the swelling of optic discs from stage 0 (no papilledema) to stage 5 (severe papilledema) [19]. PwIIH with stage 0 optic nerve swelling on the Frisén-Scale were designated as inactive IIH.

For OCT imaging, we used the same spectral-domain OCT (Spectralis OCT, Heidelberg Engineering, Heidelberg, Germany; software Heidelberg eye explorer software version 6.9a) adhering to the OSCAR-IB quality control criteria and describing findings in accordance with the APOSTEL criteria [20, 21].

For peripapillary retinal nerve fiber layer (pRNFL) measurement, a 12° (3.4 mm) ring scan centered on the optic nerve head was used (1536 A-scans, automatic real-time tracking [ART]: 100 averaged frames) [22]. Ganglion cell layer (GCL) volume was measured without pupil dilatation in both eyes of each patient by means of a 20° × 20° macular volume scan (centered on the macula with 512 A-scans and 25 B-scans aligned vertically with 16 averaged frames). Volume values characterize the mean volume of the circular area centered around the foveola, corresponding to the 6 mm ring of the circular grid defined by the Early Treatment Diabetic Retinopathy Study [23]. Image processing was semiautomated using the built-in proprietary software for automated layer segmentation and manual correction of obvious errors. Measurements of worse eyes were used for statistical analysis, i.e., higher pRNFL thickness as a marker of oedema and lower GCL volume as a marker of neuroaxonal loss.

For assessment of the optic nerve sheath diameter (ONSD), we performed transbulbar sonography (ABSolu, Quantel Medical, Cournon d’Auvergne, France) after topical anesthesia with oxybuprocaine eye drops. A B-scan with the 10 MHz probe placed temporally was used to visualize the optic nerve in horizontal and vertical sections. Presence or absence of the bat sign, a clearly differentiable bat-shaped echo-poor image of the optic nerve sheaths indicating perineural CSF congestion, was documented. We subsequently performed quantitative measurement of ONSD using standardized amplitude modulation (A-scan) echography with tissue sensitivity settings, placing the 8 MHz A-scan probe on the temporal eye equator in primary gaze position, as previously described by others [24, 25]. At least two measurements were taken within 3 mm of the posterior bulb wall, and the highest was documented as the diameter. ONSD was defined as normal (< 4.50 mm), marginal (4.50–4.99 mm), or abnormal (≥ 5.00 mm).

### Study endpoints

The primary endpoint was the percentage weight loss at M6 compared to baseline.

The secondary endpoint was the percentage weight loss at M3 compared to baseline.

Further secondary endpoints assessed at M3 and M6 comprised:

Weight

- proportion of patients with ≥ 5% weight loss
- proportion of patients with ≥ 10% weight loss
- BMI compared to baseline

Headache

- MHD compared to baseline
- 30% responder-rate (rate of patients with $\ge$ 30% reduction in MHD compared to baseline)
- 50% responder-rate (rate of patients with ≥ 50% reduction in MHD compared to baseline)
- headache freedom: < 1 MHD
- rate of patients who reverted from chronic headache (≥ 15 MHD) at baseline to episodic headache (< 15 MHD)

Ophthalmological outcomes

- degree of papilledema on Frisén scale compared to baseline
- rate of inactive IIH
- visual impairment: visual acuity ≥ 0.2 logMAR and/or < –2.0 dB in static threshold perimetry
- decrease of visual acuity by ≥ 0.2 logMAR and/or mean deviation by ≥ 2.0 dB in static threshold perimetry compared to baseline
- change in pRNFL thickness and GCL volume compared to baseline
- change in ONSD compared to baseline

Medication

- dosage of the GLP-1-RA
- adherence to the GLP-1-RA therapy
- use and dosage of acetazolamide, topiramate, and furosemide

Tolerability

- type and frequency of adverse events (AEs)
- type and frequency of severe adverse events (SAEs)
- rate of patients with any AE
- rate of patients with any SAE

AEs were reported and graded using the Common Terminology Criteria for Adverse Events (CTCAE) classification.

### Statistics

Statistical analysis was performed using SPSS 26.0 (SPSS Inc, Chicago, IL, USA). Categorical variables were expressed in absolute frequencies and percentages, continuous parametric variables as mean and standard deviation (SD) and continuous non-parametric variables as median with inter-quartile range (IQR) or absolute range (AR) as appropriate.

The primary endpoint, change of weight loss over time, was analyzed by repeated-measures ANOVA comparing patients with GLP-1-RAs plus usual care weight management (GLP-1-RA group) to patients with usual care weight management only (UCWM).

Secondary endpoints were univariately compared between the GLP-1-RA and UCWM groups.

Headache endpoints were analyzed by repeated measures linear regression models with the outcome variables as the dependent variable and GLP-1-RA + UCWM vs. UCWM (reference category) as the independent variable adjusted for headache frequency at baseline and in a second step also for weight loss to evaluate potential independent treatment effects of GLP-1-RAs.

Similarly, ophthalmological endpoints were investigated adjusting for (1) papilledema degree, (2) pRNFL thickness, (3) GCL volume, or (3) ONSD at baseline as appropriate and in a second step also for weight loss to evaluate potential independent treatment effects of GLP-1-RAs.

Predefined sensitivity analyses to determine potential confounding influence were conducted with the same statistical analysis set-up removing (1) patients with IIH without papilledema (IIH-WOP), and (2) patients with pre-existing migraine. Significance level was set at a two-sided *p*-value < 0.05 with Bonferroni correction for multiple testing.

## Results

In all, 39 pwIIH (mean age 33.6 years [SD 8.0], 92.3% female, median BMI 36.3 kg/m^2^ [IQR 31.4–38.3]) were included, with 13 pwIIH being treated with GLP-1-RAs and remaining adherent to the treatment throughout the study. Ten patients received semaglutide 1.0 mg, and one patient each received semaglutide 0.5 mg, liraglutide 1.2 mg and liraglutide 2.4 mg (maximum tolerated dose).

**Fig. 2:**
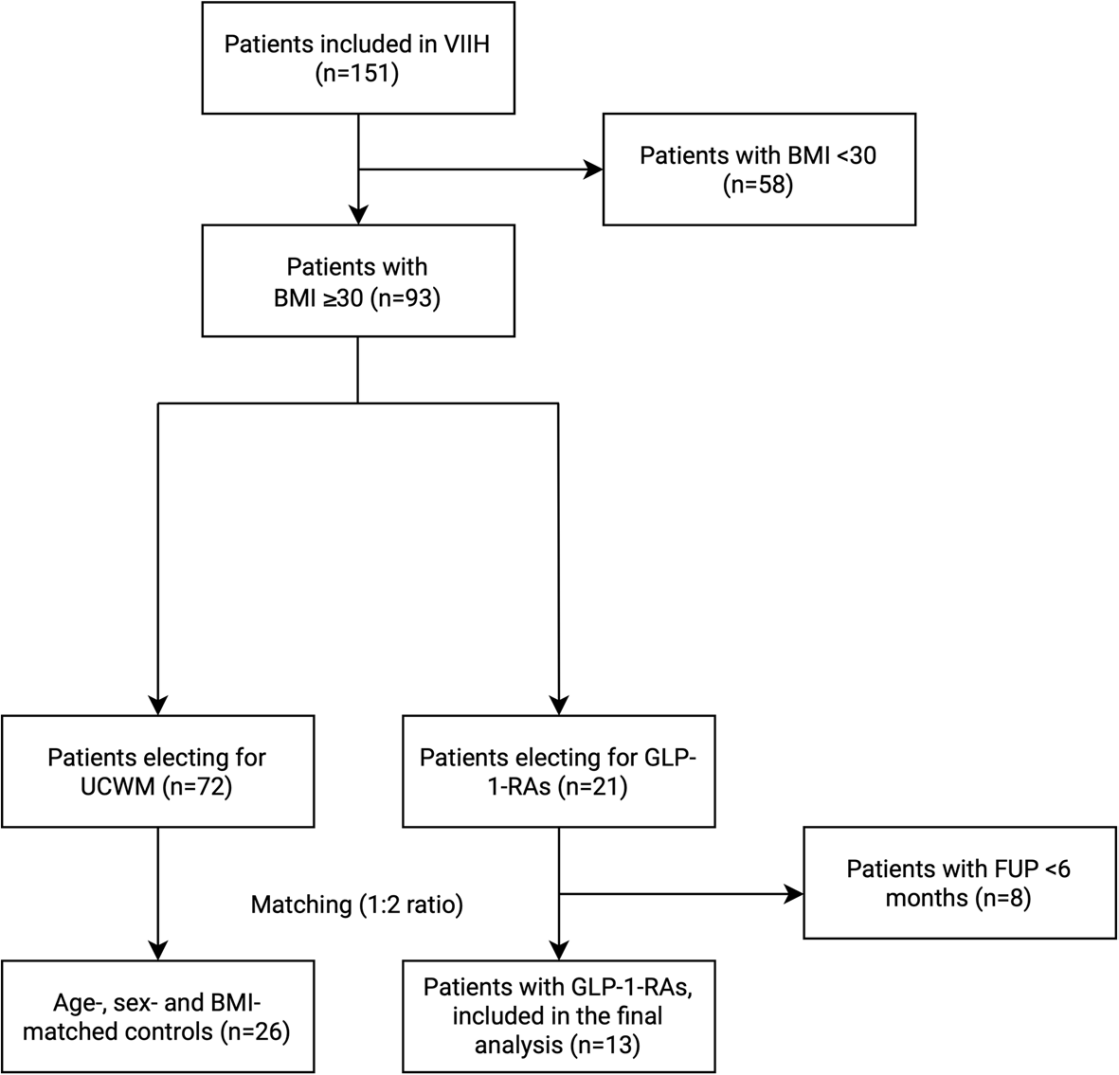
Flow chart of inclusion/exclusion process. BMI: body mass index, FUP: follow-up, GLP-1-RA: glucagon-like peptide-1 receptor agonist, UCWM: usual care weight management, VIIH: Vienna Idiopathic Intracranial Hypertension database

The inclusion/exclusion process is shown in Fig. 2. Their demographics are presented in Table 1.

**Table 1:** Demographics of the study cohort at baseline

	**GLP-1-RA group (*n* = 13)**	**UCWM (*n* = 26)**	***p*-value**
**Clinical characteristics**			
Females^a^	12 (92.3)	24 (92.3)	> 0.999
Age at baseline (years)^b^	35.1 (7.9)	32.9 (8.1)	0.435
Disease duration (weeks)^c^	40.9 (11.9–105.0)	4.0 (1.0–23.0)	**0.003**
CSF opening pressure (cmH_2_O)^b^	28.6 (7.9)	32.6 (6.1)	0.129
BMI (kg/m^2^)^c^	33.5 (31.8–39.3)	35.0 (30.4–38.1)	0.803
IIH-WOP^a^	1 (7.7)	4 (15.4)	n.a
**Therapy**			
Acetazolamide^a^	10 (76.9)	25 (96.2)	0.099
Median acetazolamide dosage (mg)^c^	1,125 (500–1,625)	750 (500–1,000)	0.602
Topiramate^a^	1 (7.7)	1 (3.8)	n.a
Topiramate dosage (mg)	100	25	n.a
Furosemide^a^	0 (0.0)	3 (11.5)	n.a
Median furosemide dosage (mg)^c^	n.a	40 (20–40)	n.a
Ventriculoperitoneal shunt	1 (7.7)	0 (0.0)	n.a
**Headache**			
Headache present^a^	12 (92.3)	15 (57.7)	**0.034**
Monthly headache days^c^	8 (3–14)	4.5 (0–12)	0.178
Chronic headache^a^	2 (15.4)	4 (15.4)	n.a
Comorbid migraine^a^	6 (46.2)	5 (19.2)	0.131
Treatment with an anti-CGRP mAb^a^	3 (23.1)	1 (3.8)	n.a
**Ophthalmological findings**			
Papilledema^a^	8 (61.5)	21 (80.8)	0.253
Frisén-Scale^d^	1 (0–3)	1 (0–4)	0.353
Visual acuity of worse eye (logMAR)^d^	1.20 (0.10–1.25)	1.20 (0.10–1.60)	0.941
Decreased visual acuity^a^	2 (15.4)	2 (7.7)	0.589
Visual field mean deviation of worse eye (dB)^c^	–1.01 (–6.55, 0.02)	–3.24 (–6.97, –1.04)	0.230
Abnormal visual field^a^	4 (30.8)	17 (65.4)	0.087
pRNFL thickness of worse eye (µm)^b^	100.9 (38.8)	115.0 (34.7)	0.254
GCL volume of worse eye (mm^3^)^c^	1.02 (0.93–1.05)	1.05 (0.94–1.13)	0.504
Presence of a bat sign^a^	10 (76.9)	25 (96.2)	0.099
Abnormal ONSD^a^	9 (69.2)	17 (65.4)	0.601
Marginal ONSD^a^	2 (15.4)	7 (26.9)	
ONSD of worse eye (mm)^b^	5.54 (1.17)	5.46 (0.81)	0.805

*Anti-CGRP mAbs* Monoclonal antibodies against calcitonin gene-related peptide, *CSF* Cerebrospinal fluid, *GCL* Ganglion cell layer, *GLP-1* Glucagon-like peptide-1, *IIH* Idiopathic intracranial hypertension, *IIH-WOP* Idiopathic intracranial hypertension without papilledema, *UCWM* Usual care weight management, *ONSD* Optic nerve sheath diameter, *pRNFL* Peripapillary retinal nerve fiber layer

^a^Number (percentage)

^b^Mean (standard deviation)

^c^Median (interquartile range)

^d^Median (range)

### Primary endpoint

**Fig. 3:**
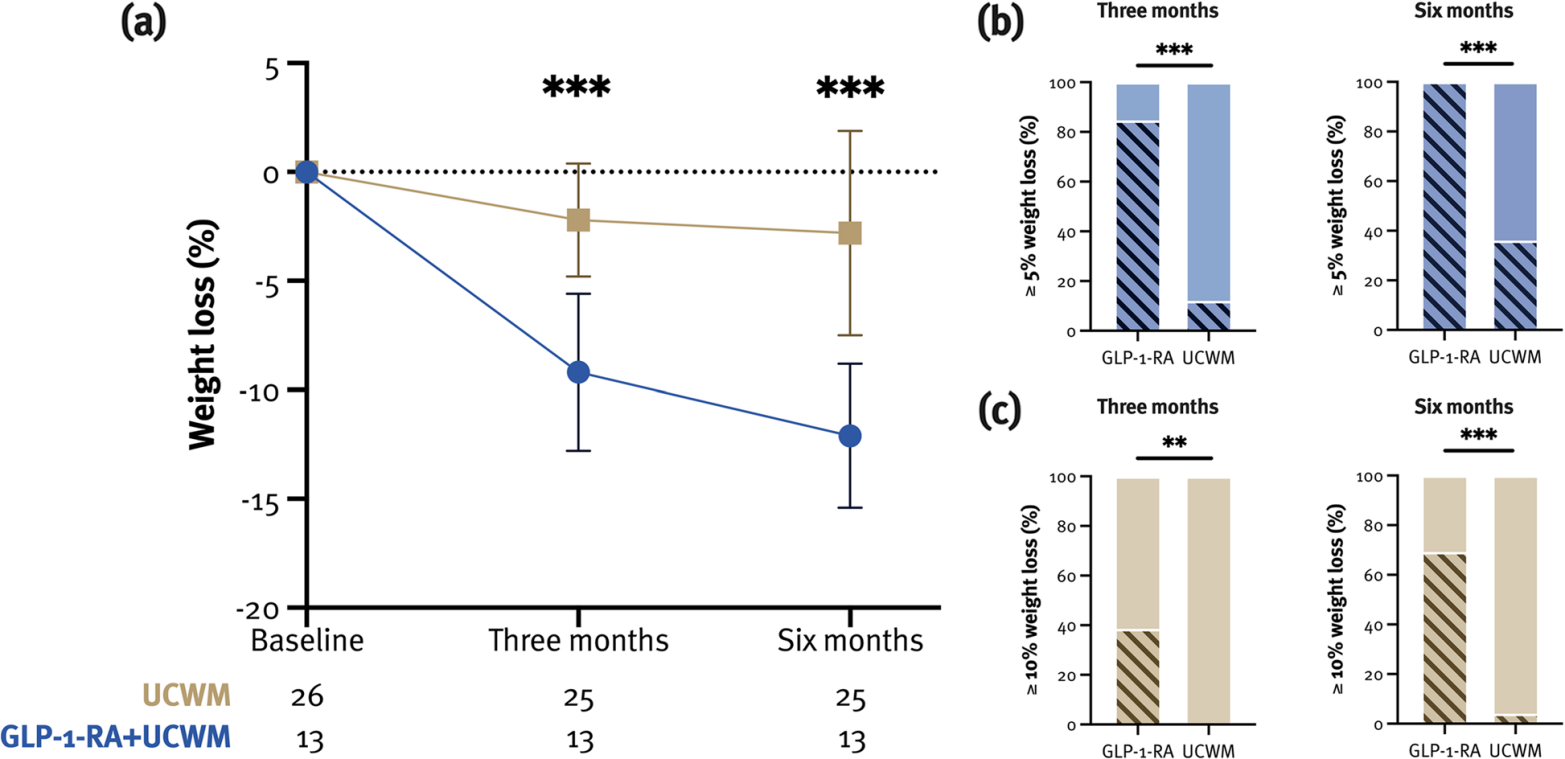
Weight loss in patients treated with GLP-1-RAs and controls. **p < 0.01, ***p < *0.001*

Percentage weight loss at M6 was statistically significantly higher in the GLP-1-RA group than in the UCWM group (–12.0% [3.3] vs. –2.8% [4.7]; *p* < 0.001) (Fig. 3).

### Secondary endpoints

#### Weight loss

A difference in weight loss was already seen at M3 (–9.2% [3.6] vs. –2.2% [2.6]; *p* < 0.001) (Fig. 3). Moreover, a higher proportion of patients treated with GLP-1-RAs had lost ≥ 5% of weight at M3 (84.6% vs. 12.0%; *p* < 0.001) and M6 (100.0% vs. 36.0%; *p* < 0.001), and the same was true for a weight loss of ≥ 10% (M3: 38.5% vs 0%, *p* = 0.003; M6: 69.2% vs. 4.0%, *p* < 0.001) (Fig. 3).

Weight loss was paralleled by a statistically significant decrease in BMI at M6 (–3.4 kg/m^2^ [2.5] vs.–1.7 kg/m^2^ [2.0]; *p* = 0.029), but not at M3 (–1.9 kg/m^2^ [1.9] vs. –1.3 kg/m^2^ [1.4]; *p* = 0.271).

#### Headache outcomes

**Fig. 4:**
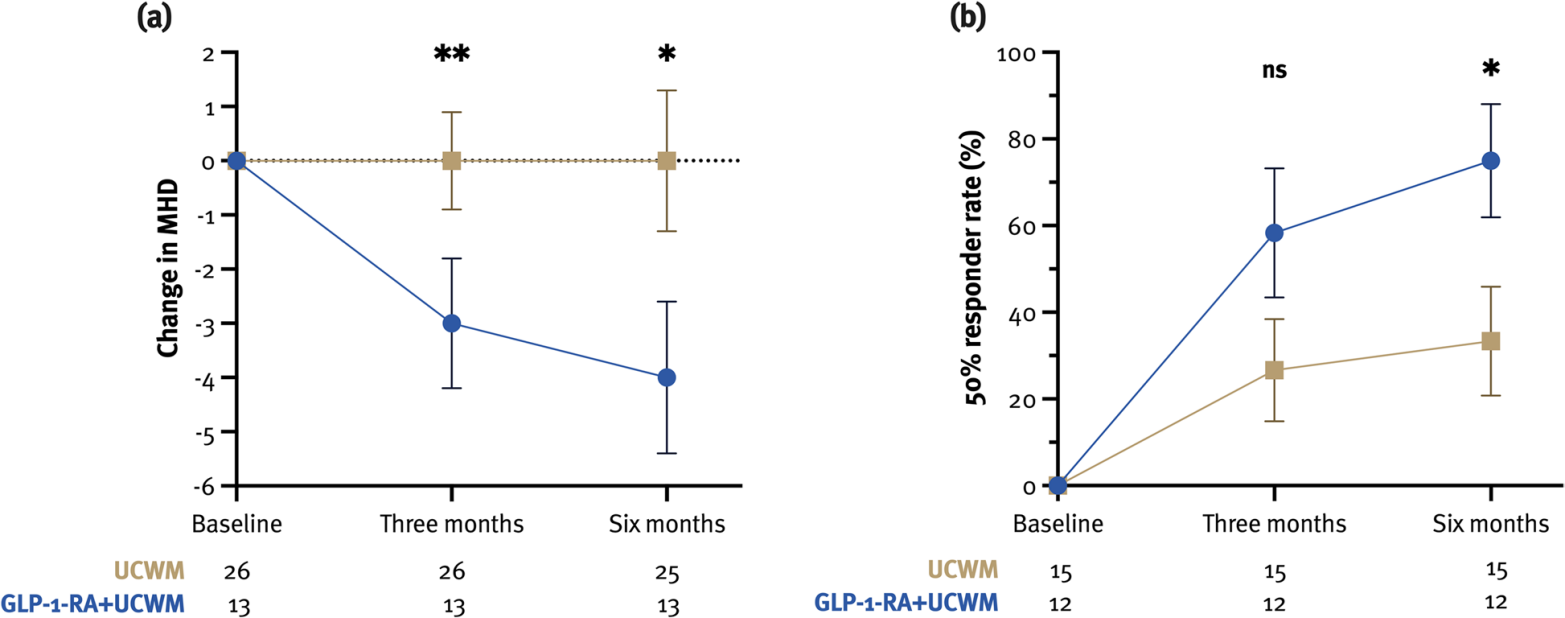
Change in monthly headache days (MHC) in patients treated with GLP-1-RAs and controls (**a**). Fifty percent responder rate at M6 was 75.0% and 33.3% in the GLP-1-RA and the UCWM group, respectively (**b**). *p < 0.05, **p < 0.01

The median reduction in MHD was significantly higher in the GLP-1-RA group at both M3 (–3 [–7.5, –1.5] vs. 0 [–2, 0]; *p* = 0.003) and M6 (–4 [–10.5, 0.5] vs. 0 [–3, 1]; *p* = 0.02) (Fig. 4). However, neither of the treatment groups was associated with the reduction in MHD, whereas MHD at baseline was (M3: $\beta$= –0.29, 95% CI –0.46, –0.11; *p* = 0.003; M6: $\beta$= –0.43, 95% CI –0.67, –0.20; *p* = 0.001) (Supplementary Table 2).

At baseline, twelve (30.8%) patients had no headache and were therefore excluded from analyses of headache improvement. Univariately, no differences in 30% and 50% responder-rate were seen between the groups (Supplementary Table 1). In a multivariate model, the GLP-1-RA group was associated with 50% responder-rate at M6 (Fig. 4), which did not withstand the adjustment for weight loss (Supplementary Table 2).

Headache freedom at M6 was achieved in 4 (30.8%) and 8 (32.0%) patients from the GLP-1-RA and UCWM group, respectively (Fig. 4, Supplementary Table 1). Headache freedom was not associated with the treatment group but with the MHD at baseline (F(1,35) = 20.99, *p* < 0.001), whereas chronic headache was associated with the UCWM group, but did not withstand the adjustment for weight loss (Supplementary Table 2).

Sensitivity analyses removing (1) IIH-WOP and (2) patients with migraine did not significantly change the overall results or impact of single variables.

### Ophthalmological outcomes

#### Papilledema

Change in Frisén scale grading was did not differ between groups (Supplementary Table 3). At M6, papilledema was still present in 7 (58.3%) and 19 (76.0%) patients from the GLP-1-RA and UCWM group (Supplementary Table 3). Rate of inactive IIH was not associated with the treatment group but with the papilledema degree at baseline (M3: $\beta$=0.38, 95% CI 0.20, 0.57; M6: $\beta$=0.43, 95% CI 0.29, 0.58; both *p* < 0.001) (Supplementary Table 4).

#### Visual impairment and visual worsening

No differences in visual impairment between groups were seen (Supplementary Table 3), with visual impairment being associated with visual impairment at baseline (M3: $\beta$=0.79, 95% CI 0.54, 1.03; M6: $\beta$=0.77, 95% CI 0.50, 1.03; both *p* < 0.001) (Supplementary Table 4). Visual worsening was seen in one (8.3%) patient from the GLP-1-RA group and two (8.0%) patients from the UCWM group at M6 (Supplementary Table 3). Visual worsening was neither associated with the treatment group nor with the papilledema degree or visual impairment at baseline (Supplementary Table 4).

#### Optical coherence tomography

Change in pRNFL thickness was not associated with the treatment group but with pRNFL thickness at baseline (M3: $\beta$= –0.32, 95% CI –0.41, –0.23; M6: $\beta$= –0.53, –0.66, –0.41; both *p* < 0.001) (Supplementary Table 4). Change in GCL was neither associated with the treatment group nor with GCL volume at baseline (Supplementary Table 4).

#### Ultrasonography

Abnormal ONSD was seen in 9 (69.2%) and 17 (65.4%) patients from the GLP-1-RA and the UCWM group, respectively. Change in ONSD at M3 and M6 was not associated with the treatment group, but with ONSD at baseline (M3: $\beta$= –0.69, 95% CI –0.99, –0.39; M6: $\beta$= –0.95, 95% CI –1.18, –0.72; both *p* < 0.001) (Supplementary Table 4).

Sensitivity analyses removing (1) IIH-WOP and (2) patients with migraine did not significantly change the overall results or impact of single variables.

### Medication for IIH

As most patients received acetazolamide, further analyses were only performed on the latter. The median acetazolamide dosage at M6 in the GLP-1-RA and UCWM group was 750 mg (250–1,500) and 750 mg (500–1,500), respectively, with a significantly higher reduction in the GLP-1-RA group (–16.5% [–50, 0] vs. 0% [–25, 50], *p* = 0.04). The GLP-1-RA group was associated with reduction in acetazolamide dosage at M6 ($\beta$= –288.15, 95% CI –553.09, –23.22, *p* = 0.03), which did not withstand the adjustment for weight loss.

### Safety

Overall, 9 (69.2%) patients treated with GLP-1-RAs reported at least one AE. Most common AEs were gastrointestinal, and of mild or moderate severity (Table 2). Increased lipase and alanine aminotransferase were noted in 3 (23.1%) and 1 (7.7%) of the patients, respectively, with none of those resulting in pancreatitis and/or cholelithiasis. No SAEs were reported, and none of the AEs led to premature treatment discontinuation.

**Table 2:** Treatment-emergent adverse events (AEs) by preferred term

	**GLP-1-RA group (*n* = 13)**
Any AEs	9 (69.2)
Mild AEs	1 (7.7)
Moderate AEs	8 (61.5)
Severe AEs	0 (0.0)
AEs leading to premature treatment discontinuation	0 (0.0)
AEs occurring in $\ge$ 5% of patients	
Nausea	9 (69.2)
Mild	1 (7.7)
Moderate	8 (61.5)
Decreased appetite	2 (15.4)
Increased lipase	3 (23.1)
Mild (< 1.5 ULN)	3 (23.1)
Diarrhea	1 (7.7)
Mild	1 (7.7)
Hypoglycemia	1 (7.7)
Mild (55–70 mg/dL)	1 (7.7)
Increased ALAT	1 (7.7)
Mild (< 3.0 ULN)	1 (7.7)
Dyspepsia	1 (7.7)
Mild	1 (7.7)

*ALAT* Alanine aminotransferase, *GLP-1* Glucagon-like peptide-1, *ULN* Upper limit of normal

## Discussion

The aim of this study was to evaluate the efficacy and safety of GLP-1-RAs in pwIIH. Three key findings result from our study: (1) pwIIH treated with GLP-1-RAs lose significantly more weight than pwIIH electing for UCWM only, (2) treatment with GLP-1-RAs is associated with favorable headache outcomes, and (3) treatment with GLP-1-RAs is not associated with any severe adverse events in IIH, and thus presents a safe treatment option for weight management in pwIIH.

Weight loss has been long recognized as a disease-modifying therapy in IIH with patients being commonly recommended to lose ≥ 10% weight, although the amount of weight loss required to achieve disease remission remains unclear [1]. Recently, bariatric surgery was proven to be superior to UCWM in lowering intracranial pressure [26], also by achieving much higher weight loss compared to UCWM. However, due to its invasiveness, it should be offered preferentially to patients with exhausted non-pharmacological and pharmacological treatment options. In our study, we show that GLP-1-RAs are superior to UCWM both with respect to the extent of weight loss and the proportion of patients achieving the recommended ≥ 10% weight loss, highlighting the potential of concomitant pharmacological treatment for weight management in IIH. Besides, treatment with GLP-1-RA was associated with reduction in acetazolamide dosage at M6, most probably due to significantly higher weight loss. However, patients receiving GLP-1-RAs had also been treated longer, which could have affected the likelihood of reducing the medication for IIH.

While IIH can be a deleterious condition leading to visual worsening or even blindness, it is also associated with reduced quality of life due to severe headache [27]. More than half of IIH patients experience persistent headache following resolution of papilledema and normalization of CSF pressure [28–30]. Furthermore, migraine poses a risk factor for headache development, and migraine-like headache being associated with lower likelihood of headache improvement and headache freedom after one year [31]. It seems that obesity plays an important role in the pathophysiology of migraine as well, with obese women having elevated plasma levels of calcitonin gene-related peptide (CGRP) compared to controls [32, 33]. In our study, GLP-1-RAs significantly reduced the (relative) number of MHD, with no patient from the GLP-1-RA group suffering from chronic headache at M6. Of note, almost half of patients treated with GLP-1-RAs also had pre-existing migraine as comorbidity (26.7% in the UCWM group), which could predispose them to worse (rather than better) headache outcomes [31], providing additional evidence of a favorable effect of GLP-1-RAs on headache improvement. After adjustment of the model for weight loss, associations between the groups and headache outcomes did not remain statistically significant although a trend towards better headache outcomes in the GLP-1-RA group was noted. In that way, headache improvement might not only be a consequence of weight loss but also another direct effect of GLP-1-RAs on intracranial pressure [15], which seems to be directly associated with MHD and headache severity [30].

In terms of vision, visual impairment can be insidious in IIH and may appear months or years after initial symptoms, being reported in up to 20% of patients [34]. However, studies have shown that retinal layer thickness is significantly lower in pwIIH compared to healthy controls, meaning that axonal loss is more widespread than previously suspected, and also occurs in apparently effectively treated pwIIH and/or patients not reporting any visual disturbances at all [35]. In our cohort, visual impairment at baseline was seen in 21 (53.8%) patients and remained the only predictive factor of visual impairment but not visual worsening at M6.

In pwIIH, papilledema can be detected and monitored by measuring the pRNFL thickness. The latter increases in papilledema as a result of axoplasmic statis in the swollen retinal ganglion cells [36–38]. In our cohort, the majority of patients exhibited active IIH, especially in the UCWM group with significantly shorter disease duration, with slightly higher yet not significant change in the pRNFL thickness in this group. Measurement of GCL volume or thickness is not affected by axonal swelling and allows for a more accurate quantification of neuroaxonal damage during acute exacerbation of IIH compared to the pRNFL thickness. In our cohort, GLP-1-RAs did not show a significant effect on GCL volume loss during the first six months. Most probably, the observation period of six months was too short to sensitively detect worsening in visual outcomes, including GCL volume loss, also proven by a recent study in which a delayed decline in visual field and GCL volume after 12 months was seen [39].

Clinical trials have shown that semaglutide and liraglutide are generally well tolerated, with gastrointestinal disorders being the most commonly reported AEs, including nausea, diarrhea, vomiting, and constipation, leading to dose reduction or temporary treatment interruption in 12.5% of study participants [40–42]. They are most prevalent during or shortly after dose escalation and decrease afterwards. While no SAEs were seen in our cohort, some complained about mild gastrointestinal AEs. Although nausea was commonly classified as moderate due to its associated loss of appetite and significant weight loss, this was rather an expected effect than a true AE and also a reason why patients with IIH have been offered to be treated with GLP-1-RAs in the first place. Besides, only in a minority of patients, elevation of pancreatic or liver enzymes was seen, resulting in no pancreatitis and/or cholelithiasis occurrence, which was also verified in a recent meta-analysis showing that treatment with GLP-1-RAs is not associated with increased risk for acute pancreatitis, renal failure, or malignant neoplasms [43]. From this perspective, GLP-1-RAs appear as a safe treatment option. Moreover, they also have a positive effect on cardiometabolic risk factors most probably translating to a moderate reduction in the risk of major adverse cardiovascular events that still remain undetermined. As we did not observe any discontinuation of treatment during the study period, it remains unclear whether pwIIH successfully treated with GLP-1-RAs sustain their weight loss, or a ‘yo-yo effect’ occurs [44], which would pose an increased risk for a disease relapse.

There are some limitations acknowledged to this study, including a relatively low sample size (owing to the rarity of IIH) and a short follow-up period, partially mitigated by the standardized data collection and thorough quality control applied within the VIIH. Besides, our study was neither randomized nor blinded, leading to potential bias, whose influence should be mitigated by matching for relevant confounders. Moreover, treatment groups significantly differed in disease duration at baseline as most patients have not decided for the treatment with GLP-1-RAs until having failed to achieve significant weight loss on their own, which could have affected our results. Also, intercurrent events were not taken into consideration, such as smoking cessation, which could have affected weight loss [45]. While a 6-month follow-up period is commonly used to determine outcome of therapeutic interventions in IIH studies, we intend to further follow up the patients and also recruit new ones to obtain more long-term data. Treatment regimens followed best practice recommendations but naturally varied inter-individually, inducing potential bias. As only one patient with IIH-WOP was treated with a GLP-1-RA, this was insufficient to conduct subgroup analyses, so we could only perform sensitivity analyses removing IIH-WOP patients to exclude potential confounding effect on our results.

## Conclusions

In conclusion, GLP-1-RAs present a safe, effective and well-tolerated treatment option for achieving significant weight loss in pwIIH, leading to headache improvement after six months as well. The short-term study design was insufficient to prove their effect on visual outcomes. Longer observational periods with larger cohorts are needed to further elucidate the role of GLP-1-RAs in treating IIH.

## Supplementary Information

**Additional file 1.**

## Data Availability

Data supporting the findings of this study are available from the corresponding author upon reasonable request by a qualified researcher and upon approval by the data-clearing committee of the Medical University Vienna.

## Abbreviations

AE: Adverse event
AgRP: Agouti-related peptide
ALAT: Alanine aminotransferase
Anti-CGRP mAbs: Monoclonal antibodies against calcitonin gene-related peptide
AP: Area postrema
BMI: Body mass index
CART: Cocaine- and amphetamine-regulated transcript
CGRP: Calcitonin gene-related peptide
CSF: Cerebrospinal fluid
ENS: Enteric nervous system
FUP: Follow-up
GCL: Ganglion cell layer
GLP-1: Glucagon-like peptide-1
GLP-1-RA: Glucagon-like peptide-1 receptor agonist
Hyp: Hypothalamus
ICP: Intracranial pressure
IIH: Idiopathic intracranial hypertension
IIH-WOP: Idiopathic intracranial hypertension without papilledema
MHD: Monthly headache days
NPY: Neuropeptide Y
NTS: Nucleus tractus solitarii
OCT: Optical coherence tomography
ONSD: Optic nerve sheath diameter
POMS: Proopiomelanocortin
pRNFL: Peripapillary retinal nerve fiber layer
pwIIH: Patients with idiopathic intracranial hypertension
SFO: Subfornical organ
UCWM: Usual care weight management
ULN: Upper limit of normal
VIIH: Vienna Idiopathic Intracranial Hypertension
