# Supplementary material for: Treatment with GLP-1 receptor agonists is associated with significant weight loss and favorable headache outcomes in idiopathic intracranial hypertension
Source: J Headache Pain. 2023 Jul 18;24(1):89. doi: 10.1186/s10194-023-01631-z (PMC10353241; doi:10.1186/s10194-023-01631-z)
Supplement: Supplementary file 1 — Additional file 1. [file 10194_2023_1631_MOESM1_ESM.docx]

**Supplementary Table 1.** Univariate analyses of headache outcomes.

|  | **Three months** | | | **Six months** | | |
| --- | --- | --- | --- | --- | --- | --- |
|  | **GLP-1-RA group (n=13)** | **UCWM (n=26)** | ***p*-value** | **GLP-1-RA group (n=13)** | **UCWM (n=25)** | ***p*-value** |
| MHD^a^ | 4 (0–6) | 2 (0–6.5) | 0.670 | 3 (0–5) | 3 (0–7) | 0.671 |
| Absolute change in MHC^a^ | –3 (–7.5, –1.5) | 0 (–2, 0) | **0.003** | –4 (–10.5, –0.5) | 0 (–3, 1) | **0.021** |
| Relative change in MHC^a^ | –57 (–90.5, –15) | 0 (–23.3, 0) | **0.002** | –71 (–96, –4.5) | 0 (–33, 10) | **0.005** |
| 30% responder-rate^b,†^ | 9 (75.0) | 7 (46.7) | 0.239 | 9 (75.0) | 8 (53.3) | 0.424 |
| 50% responder-rate^b,†^ | 7 (58.3) | 4 (26.7) | 0.130 | 9 (75.0) | 5 (33.3) | 0.054 |
| Headache freedom^b^ | 4 (30.8) | 12 (46.2) | 0.495 | 4 (30.8) | 8 (32.0) | >0.999 |
| Chronic headache^b^ | 0 (0.0) | 3 (11.5) | 0.538 | 0 (0.0) | 4 (16.0) | 0.278 |

GLP-1-RA: glucagon-like peptide-1 receptor agonist, MHD: monthly headache days, UCWM: usual care weight management

^a^Median (interquartile range), ^b^Number (percentage)

^†^In the responder-rate analyses, only patients with headache at baseline were involved (12 and 15 patients from the GLP-1-RA and UCWM group, respectively)

**Supplementary Table 2.** Linear regression models for headache outcomes.

|  | **Three months** | | **Six months** | |
| --- | --- | --- | --- | --- |
|  | $\boldsymbol{\beta}$ **(95% CI)** | ***p*-value** | $\boldsymbol{\beta}$ **(95% CI)** | ***p*-value** |
| Absolute change in MHC | –2.36 (–5.12, 0.39) | 0.091 | –3.57 (–7.21, 0.07) | 0.055 |
| Relative change in MHC | –41.60 (–72.22, –10.99) | **0.009** | –57.60 (–99.18, –16.02) | **0.008** |
| After adjustment for weight loss | –39.63 (–87.70, 8.44) | 0.103 | –35.27 (–95.46, 24.93) | 0.242 |
| 30% responder-rate^†^ | 0.38 (–0.11, 0.67) | 0.147 | 0.22 (–0.17, 0.61) | 0.264 |
| 50% responder-rate^†^ | 0.33 (–0.07, 0.73) | 0.104 | 0.44 (0.05, 0.83) | **0.027** |
| After adjustment for weight loss | Not retained | n.a. | 0.46 (–0.08, 0.99) | 0.094 |
| Headache freedom | –0.06 (–0.33, 0.21) | 0.639 | 0.04 (–0.24, 0.33) | 0.763 |
| Chronic headache | –0.16 (–0.30, –0.02) | **0.027** | –0.20 (–0.37, –0.03) | **0.026** |
| After adjustment for weight loss | –0.13 (–0.35, 0.09) | 0.232 | –0.22 (–0.47, 0.04) | 0.096 |

MHC: monthly headache days

^†^In the responder-rate analyses, only patients with headache at baseline were involved (12 and 15 patients from the GLP-1-RA and UCWM group, respectively)

**Supplementary Table 3.** Univariate analyses of visual outcomes.

|  | **Three months** | | | **Six months** | | |
| --- | --- | --- | --- | --- | --- | --- |
|  | **GLP-1-RA group (n=13)** | **UCWM (n=24)** | ***p*-value** | **GLP-1-RA group (n=12)** | **UCWM (n=25)** | ***p*-value** |
| Papilledema^a^ | 8 (61.5) | 19 (82.6) | 0.235 | 7 (58.3) | 19 (76.0) | 0.443 |
| Frisén-Scale^b^ | 1 (0–2) | 1 (0–3) | 0.603 | 1 (0–2) | 1 (0–3) | 0.761 |
| Visual acuity of worse eye (logMAR)^b^ | 1.20 (0.10–1.20) | 1.20 (0.10–1.60) | 0.649 | 1.20 (0.02–1.25) | 1.20 (0.10–1.25) | 0.643 |
| Decreased visual acuity^a^ | 2 (15.4) | 2 (8.3) | 0.602 | 2 (16.7) | 3 (12.0) | >0.999 |
| Visual field mean deviation of worse eye (dB)^c^ | –6.23 (11.04) | –4.66 (6.82) | 0.987 | –6.11 (11.00) | –4.64 (5.60) | 0.402 |
| Abnormal visual field (perimetry)^a^ | 3 (23.1) | 11 (50.0) | 0.162 | 4 (36.4) | 11 (47.8) | 0.715 |
| Visual worsening^a^ | 1 (7.7) | 4 (18.2) | 0.630 | 1 (8.3) | 2 (8.0) | >0.999 |
| pRNFL thickness of worse eye (µm)^d^ | 96.6 (35.9) | 102.3 (23.5) | 0.667 | 90.6 (29.0) | 99.4 (18.6) | 0.023 |
| Mean change in pRNFL thickness of worse eye (µm)^d^ | –4.4 (12.2) | –13.7 (16.6) | **0.044** | –10.5 (27.4) | –16.6 (23.0) | 0.603 |
| GCL volume of worse eye (mm^3^)^c^ | 1.02 (0.92–1.08) | 1.04 (0.96–1.13) | 0.649 | 1.03 (0.91–1.05) | 1.02 (0.96–1.15) | 0.540 |
| Median change in GCL volume of worse eye (mm^3^)^c^ | 0.01 (–0.03, 0.02) | –0.01 (–0.03, 0.00) | 0.181 | –0.01 (–0.04, 0.01) | –0.01 (–0.02, 0.00) | 0.856 |
| Presence of a bat sign^a^ | 8 (61.6) | 21 (87.5) | 0.100 | 9 (75.0) | 19 (79.2) | >0.999 |
| Abnormal ONSD^a^ | 6 (46.2) | 15 (62.5) | 0.489 | 5 (50.0) | 14 (58.3) | 0.718 |
| ONSD of worse eye (mm)^d^ | 4.78 (0.97) | 5.24 (0.78) | 0.204 | 4.95 (0.51) | 5.24 (0.67) | 0.520 |
| Mean change in ONSD of worse eye (mm)^d^ | –0.93 (1.03) | –0.16 (1.05) | 0.132 | –0.76 (1.15) | –0.15 (1.13) | 0.293 |

GCL: ganglion cell layer, GLP-1-RA: glucagon-like peptide-1 receptor agonist, UCWM: usual care medical weight management, ONSD: optic nerve sheath diameter, pRNFL: peripapillary retinal nerve fiber layer

^a^Number (percentage), ^b^Median (range), ^c^Median (interquartile range), ^d^Mean (standard deviation)

**Supplementary Table 4.** Linear regression models for visual outcomes.

|  | **Three months** | | **Six months** | |
| --- | --- | --- | --- | --- |
|  | $\boldsymbol{\beta}$ **(95% CI)** | ***p*-value** | $\boldsymbol{\beta}$ **(95% CI)** | ***p*-value** |
| Change in fundoscopy on Frisén scale | 0.11 (–0.28, 0.50) | 0.566 | 0.06 (–0.25, 0.36) | 0.713 |
| Rate of inactive IIH | –0.04 (–0.36, 0.27) | 0.774 | –0.10 (–0.35, 0.15) | 0.409 |
| Visual impairment | –0.18 (–0.56, 0.21) | 0.353 | –0.22 (–0.61, 0.18) | 0.272 |
| Visual worsening | –0.04 (–0.29, 0.21) | 0.752 | 0.09 (–0.37, 0.19) | 0.509 |
| Mean change in pRNFL thickness (µm) | 4.59 (–2.59, 11.78) | 0.202 | –1.87 (–12.13, 8.40) | 0.713 |
| Mean change in GCL volume (mm^3^) | 0.01 (–0.01, 0.02) | 0.470 | 0.01 (–0.02, 0.02) | 0.831 |
| Mean change in ONSD (mm) | –0.56 (–1.18, 0.07) | 0.080 | –0.32 (–0.81, 0.18) | 0.199 |

GCL: ganglion cell layer, IIH: idiopathic intracranial hypertension, ONSD: optic nerve sheath diameter, pRNFL: peripapillary retinal nerve fiber layer
